# Effects of total mixed ration with various silage on growth performance, serum parameters, ruminal fermentation, and bacteria community profile in beef cattle

**DOI:** 10.1002/fsn3.2498

**Published:** 2021-09-12

**Authors:** Xia Zhang, Hucheng Wang, Xusheng Guo

**Affiliations:** ^1^ State Key Laboratory of Grassland Agro‐Ecosystems Key Laboratory of Grassland Livestock Industry Innovation Ministry of Agriculture and Rural Affairs College of Pastoral Agriculture Science and Technology Lanzhou University Lanzhou China; ^2^ State Key Laboratory of Grassland Agro‐Ecosystems School of Life Sciences Lanzhou University Lanzhou China

**Keywords:** beef cattle, growth performance, microbial community, ruminal fermentation, silage

## Abstract

The possibilities of using high‐quality forages in incorporation with total mixed ration (TMR) during the fattening period of beef cattle have been investigated. A total of 30 Simmental bulls (438.94 ± 11.45 kg) were selected and randomly divided into two groups, TMR with single corn silage (SS) and TMR with various silage (MS). The whole experiment consisted of 15 days preparation period and 69 days experimental period. Rumen fluid and blood samples were taken from six beef cattle per treatment at the end of the experiment. The results showed that the average daily gain of the MS group (1.56 kg/day) was higher than (*p* < .05) the SS group (1.30 kg/day), and a decrease of feed conversion ratio in the MS (10.83) group was observed compared with SS group (12.36) (*p* < .05). The concentration of total volatile fatty acids for MS group was greater than (*p* < .05) the SS group. The activities of total antioxidant capacity and superoxide dismutase from MS group were also higher than the SS group, but lower urea nitrogen was found in the MS group from serum (*p* < .05). In addition, the abundances of the *Prevotella‐1* and *Verrucomicrobia* were higher in the MS group than the SS group (*p* < .05). An increase in the flavonoid biosynthesis was detected in the MS group compared with the SS group by Kyoto Encyclopaedia of Genes and Genomes analysis. The present findings suggest that it is economical and healthy to substitute high‐quality forage +low level of concentrate for a relatively low proportion forage +high level of concentrate in a finishing diet of beef cattle, which was a feasible and healthy strategy in the intensive feeding system.

## Graphical abstract

We investigated the effects of total mixed ration (TMR) with various silage on growth performance, serum parameters, ruminal fermentation, and microbial profiles of beef cattle. The results showed that the beef cattle fed TMR with various silage was a feasible strategy in the intensive feeding system. High‐quality forage can be used in beef cattle diets instead of concentrates, and no negative effect on growth performance and rumen fermentation of beef cattle.

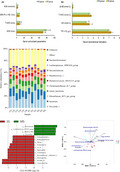

## 1 INTRODUCTION

In recent years, by the promotion of the government, the plantation of forages (alfalfa, oats, corn, and sweet sorghum) has been widely implemented leading to the production of high‐quality forage in China, bringing in rich forage resources to herbivorous animals. Additionally, many studies have proved that forage ensiled has a better nutritional value and longer storage time due to the fermentation of lactic acid bacteria (Ke et al., 2018; Zhao et al., 2019). The development of animal husbandry has focused on expanding the use of forage resources, reducing feeds cost, and increasing economic benefits. As a result, the high‐quality forages instead of concentrate is becoming increasingly popular in herbivore diets, decreasing the disadvantages of many metabolic diseases and high feed costs related to high concentrate diets in ruminant production (Alstrup et al., 2016; Marques et al., 2016; Schwaiger et al., 2013; Touno et al., 2013). The previous result found that the feeding of relatively high‐quality roughage could improve animal intake (Keady et al., 2007).

Furthermore, the traditional feeding mode (single silage or straw forage added concentrate) was used in most beef cattle farming, which limited the efficient use of forage resources (Keady et al., 2007). Thus, it is very necessary to study efficient and high‐quality feeding strategies for beef cattle. Interestingly, some studies have revealed that compared to alfalfa hay alone as a starter feed for postweaning calves, alfalfa and oat hay mixed feeding can improve dietary nitrogen efficiency and growth performance by impacting rumen fermentation, which was a better feeding strategy (Contreras‐Govea et al., 2011; Zou et al., 2018). In the context of these studies and extrapolation of in vitro study data (unpublished; Zhang, Li, Zhou, Wang), it is hypothesized that total mixed ration (TMR) with various forage silage had a positive combination effect on rumen bacteria, rumen fermentation, and growth performance of fattening beef cattle compared with a TMR with single corn silage. To verify this hypothesis, the present study investigated the effects on rumen fermentation characteristics, rumen bacteria profiles, serum parameters and growth performance of beef cattle fed TMR with alfalfa silage, oat silage, corn silage, and TMR with corn silage.

## 2 MATERIALS AND METHODS

### 2.1 Experimental area

The experimental site (North latitude 35 ′07′34″, east longitude 104 ″59′23″, altitude 1,899 m) belongs to the typical place of the semi‐arid and hilly area of the Loess Plateau, with an average annual temperature from 5.7 to 7.7°C, frost‐free period only 122–160 days, with an average annual rainfall from 300 to 400 mm. The growing season for forage is short, only 120–180 day of the year, and droughts are common. More than 3 million acres of artificial forage were planted as alfalfa, corn, oat, and sorghum (*Sorghum bicolor* L.) et al. The annual yield of silage forage was 2.69 million tons (Table 1).

**TABLE 1:** The routine nutritive components contents of different forage

Item	Corn silage	Oat silage	Alfalfa silage	Alfalfa hay	Wheat straw
Dry matter (g/kg)	315.6	367.8	339.3	883.2	899.2
Crude protein (g/kg, DM)	80.6	85.6	162.7	119.0	23.3
Neutral detergent fiber (g/kg, DM)	476.5	523.8	465.6	510.7	766.5
Acid detergent fiber (g/kg, DM)	273.4	301.3	364.6	396.5	475.4
Ash (g/kg, DM)	76.3	75.3	164.7	76.7	36.2
Ca (g/kg, DM)	18	4.6	23.1	29.2	1.6
P (g/kg, DM)	2.1	3.1	6.1	8.4	0.8

### 2.2 Animals, diets, and experimental design

The ingredient and nutrient levels of two types of diets are shown in Table 2. Thirty Simmental bulls (438.94 ± 11.45 kg) with similar age (12 months) were used in a single factor design. Animals were assigned randomly within‐pair to one of two treatments (15 animals per group). The corn silage (SS)‐based diets were composed of corn silage (40%), alfalfa hay (8%), and 52% concentrate supplement. The various silage (MS)‐based diets were composed of corn silage (27%), oat silage (15%), alfalfa silage (10%), wheat straw (10%), and 38% concentrate supplement. The TMR was formulated according to NRC (2000) recommendations for beef cattle. All animals of each group were fed in a 100‐m^2^ house, each animal lived in a separate shed, and can only touch its diets, and fed twice daily (0700 and 1700 hr) considering 5%–10% feed refusals and water was supplied ad libitum. There was 15 days adaptation period and the experimental period lasted 69 days. The daily feed intake was recorded, and the average intake during the experiment period was calculated. Meanwhile, the animals were weighed using the Electronic scale (SCS) at the beginning and end of the experiment period, and calculated the average daily gain (ADG) and feed conversion rate (FCR).

**TABLE 2:** Composition and nutrient levels of experimental diets

Item	Groups^a^	
	SS	MS
Ingredients (% DM)		
Corn silage	40.00	27.00
Oat silage		15.00
Alfalfa silage		10.00
Wheat straw		10.00
Alfalfa hay	8.00	
Corn	31.00	20.00
Wheat bran	9.00	9.00
Soybean meal	3.00	1.00
Linseed meal	5.00	5.00
NaHCO_3_	0.80	0.80
CaHPO_4_	0.40	0.40
NaCl	0.20	0.20
Premix^b^	2.50	2.50
Nutrient level, DM		
Metabolizable energy (MJ/kg)^c^	13.34	13.32
Crude protein (%)	12.89	12.86
Ca (%)	0.85	0.94
P (%)	0.71	0.76
Neutral detergent fiber (%)	32.77	38.89
Acid detergent fiber (%)	17.39	19.61

^a^ SS group: TMR with 40% single corn silage, MS group: TMR with various silage (27% corn silage, 10% alfalfa silage, and 15% oat silage).

^b^ Premix provided the following per kg of the diet: VA 160 KIU, VD_3_ 50 KIU, VE 900 IU, VB_1_ 120 mg, nicotinic acid 500 mg, Fe 1,200 mg, Cu 150 mg, Zn 1,000 mg, Mn 500 mg.

^c^ The metabolizable energy was calculated.

### 2.3 Diets and rumen samples collection

The TMR diets were sampled once a week and stored at −20°C until analysis of nutrient components of diets.

Rumen fluids were taken from six beef cattle per treatment using an esophageal catheter (Colibo A1164K) at feeding before the end of the experiment reference to Zhou et al. (2012), and then were filtered by four layers using a medical gauze. The pH values were measured immediately using a portable pH meter (pHS‐3C). Then, a part of the ruminal fluid was stored at −80°C for DNA extraction, the other parts of extracts were centrifuged at 10,000 *g* for 15 min at 4°C, the supernatants approximately 10 ml were collected and stored at −20°C for subsequent analysis of the concentrations of volatile fatty acids (VFA) profiles and ammonia nitrogen (NH_3_‐N).

The nutrient levels of experimental diets were analyzed following AOAC (1995) method. The VFA concentration of rumen fluid was measured with a gas chromatograph as described by Li et al. (2017). The NH_3_‐N concentration of rumen fluid was determined by phenol‐sodium hypochlorite colorimetry (Zhou et al., 2015).

### 2.4 Collection of blood samples and determination of indicators

Ten ml blood samples were collected from six beef cattle per treatment using the jugular vein at feeding before the end of the experiment and centrifuged at 3,500 *g* for 10 min at 4°C to separate the serum, then was kept frozen at −20°C for subsequent analysis. Serum samples were analyzed for malondialdehyde (MDA), glutathione peroxidase (GSH‐Px), total antioxidant capacity (T‐AOC), superoxide dismutase (SOD), β‐Hydroxybutyric acid (β‐HB), total cholesterol (T‐CHO), urea nitrogen (UN), and total protein (TP) activity using commercial kits (Nanjing Jiancheng Bioengineering Institute) strictly according to the manufacturer's instructions.

### 2.5 DNA extracting and sequencing analysis

The rumen fluid DNA was extracted from 300 ml of the sample, following DNA extraction kit (EZNA DNA Stool Mini) method. Each sample was extracted three times for microbial DNA, which was tested for quality and concentration, and then repeatedly mixed for 16S rRNA sequencing analysis. Bacterial amplicons were sequenced with the Illumina MiSeq platform at BMK Cloud (www.biocloud.net). Briefly, the (V3‐V4) region of the bacterial 16S rRNA was amplified. The amplification primers: Forward primer (5′‐ACTCCTACGGGAGGCAGCA‐3′) and Reverse primer (5′‐GGACTACHVGGGTWTCTAAT‐3′). A 10 μl amplification system (including 0.5 μl of full‐type gold enzyme, and 2 μl of stock solution, 1 μl of each primer, 2 μl of DNA template, and 3.5 μl of ddH_2_O_2_) was used to measure sample quality. Amplification procedure: PCR amplification procedure includes initial denaturation at 98°C for 2 min, then 25 cycles at 98°C for 30 s, 50°C for 30 s, and 72°C for 1 min, finally last extension at 72°C for 5 min. PCR products were electrophoresed on a 1.8% (w/v) agarose gel. This band was separated and purified using a gel extraction kit (TAKARA).

FLASH v1.2.7 software was used to stitch the reads of each sample to obtain raw tags, and Trimmomatic v0.33 software was used to filter the stitched raw tags to obtain clean tags. UCHIME v4.2 software (version 10.0, http://drive5.com/uparse/) was used to obtain effective tags. Using UCLUST (Edgar, 2010) in QIIME (Caporaso et al., 2010) (version 1.8.0) software, the tags were clustered at a similarity level of 97% to obtain the operational taxonomic unit (OTU). The sequencing data were classified and annotated based on the Silva taxonomy base (http://www.arb‐silva.de). Alpha index analysis was carried out using Mothur version v.1.30 (http://www.mothur.org/). LEfSe (http://huttenhower.sph.harvard.edu/lefse/) was used to analyze the microbial differences between the two treatments. When the LDA value was ≥2, the species was considered as a biomarker. The PICRUSt software (Zhang et al., 2015) was used to perform functional annotation and prediction of OTUs using the Kyoto Encyclopaedia of Genes and Genomes (KEGG) functional database. Sequencing data have been uploaded to the Sequence Read Archive of NCBI and can be accessed through the login number PRJNA608628.

### 2.6 Data analysis

The rumen bacteria sequence data were analyzed by R language, *p* < .05 as the criterion for significance. The data of rumen fermentation parameters and growth performance were analyzed using the general linear model of SPSS 19.0 software (SPSS Inc.). The general linear model of statistical analysis is as follows:

$$Y_{\mathrm{ij}}\;=\;\mu \;+\;\tau_{i}\;+\;e_{\mathrm{ij}}.$$

*Y_ij_*: *j*th observation of the *i*th treatment, *μ*: overall average, *τ_i_*: fixed effect of the *i*th treatment, and *e_ij_*: random effect. *T*‐test was used to analyze the differences between the treatments, *p* < .05 was considered statistically significant, and the trend was 0.05 ≤ *p* < .1.

## 3 RESULTS

### 3.1 Growth performance

The animal performance measured in 30 beef cattle is illustrated in Table 3. In both groups, results revealed average daily feed intake (ADFI) and ADG of MS group were greater (*p* < .05) than the SS group. The ADG of the MS group (1.56 kg/day) was increased by 20% compared with the SS group (1.30 kg/day), and the FCR of the MS group (10.96) was decreased by 12.77% compared with the SS group (12.36).

**TABLE 3:** Effects of TMR with various forage silage on growth performance in beef cattle

Item	Groups^a^		*SEM*	*p*‐Value
	SS	MS		
Initial body weight, kg	440.15	437.73	10.45	.23
Final body weight, kg	530.54	545.37	10.40	.08
Average daily gain, kg/day	1.31	1.56	0.01	.04
Average daily feed intake, DM, kg/day	16.19	16.90	0.11	.04
Feed conversion ratio^b^ (kg feed/kg weight)	12.36	10.96	0.12	.004

^a^ SS group: TMR with 40% single corn silage, MS group: TMR with various silage (27% corn silage, 10% alfalfa silage, and 15% oat silage).

^b^ Feed conversion ratio (FCR): Average daily feed intake/Average daily gain.

### 3.2 Serum biochemical and antioxidant parameters

**FIGURE 1:**
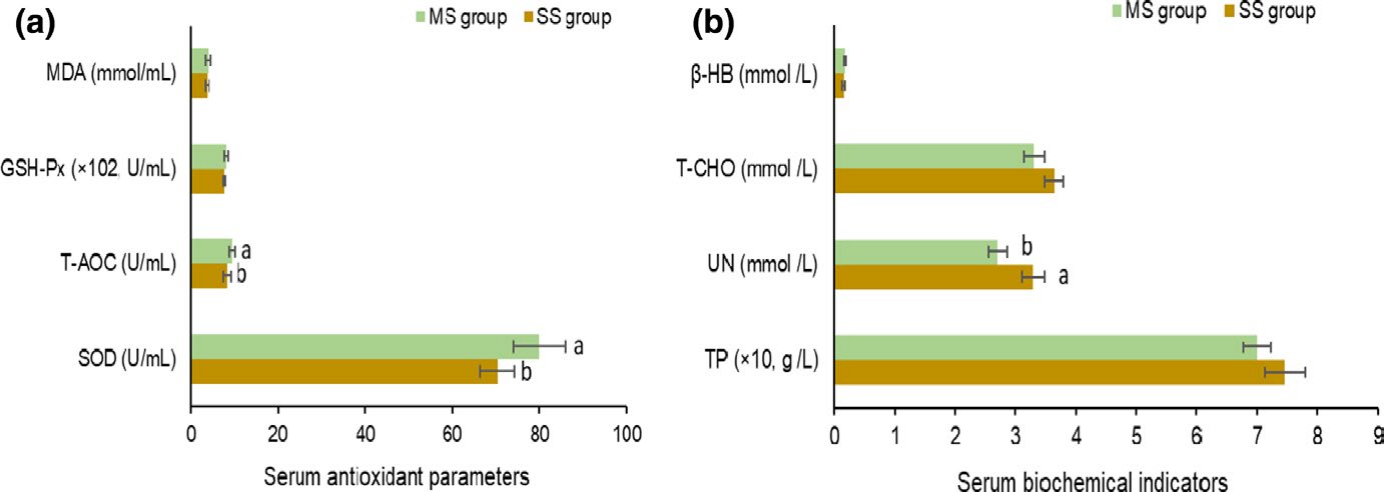
Serum antioxidant and biochemical parameters of beef cattle with different diets treatments. GSH‐Px, glutathione peroxidase; MDA, malondialdehyde; SOD, superoxide dismutase; T‐AOC, total antioxidant capacity; T‐CHO, total cholesterol; TP, total protein; UN, urea nitrogen; β‐HB, β‐Hydroxybutyric acid

The different treatments did not affect serum concentrations of MDA, GSH‐Px, β‐HB, T‐CHO, and TP (Figure 1). However, the concentrations of T‐AOC and SOD of MS group were higher than the SS group (*p* < .05), while the UN concentration was lower in the MS group (*p* < .05).

### 3.3 Rumen fermentation parameters

The results of the rumen fermentation parameters are illustrated in Table 4. The SS and MS groups had similar ruminal pH value and NH_3_‐N concentration. However, concentrations of total volatile fatty acid (TVFA), acetate, butyrate, isovalerate of the MS group were greater than the SS group (*p* < .05). The TVFA concentrations were 62.49 and 56.09 mmol/L in the MS group and SS group, respectively.

**TABLE 4:** Effects of TMR with various forage silage on rumen fermentation parameters in beef cattle

Item	Groups^b^		*SEM*	*p*‐Value
	SS	MS		
pH	7.04	7.00	0.14	.81
Ammonia nitrogen (mg/dl)	14.88	14.57	0.68	.79
Total volatile fatty acids (mmol/L)	56.09	62.49	6.87	.007
Acetate (mmol/L)	42.31	47.52	5.02	.005
Propionate (mmol/L)	10.47	10.70	1.44	.12
Isobutyric acid (mmol/L)	0.55	0.64	0.10	.01
Butyrate (mmol/L)	1.97	2.82	0.38	.001
Isovaleric acid (mmol/L)	0.33	0.50	0.08	.008
Valerate (mmol/L)	0.29	0.31	0.11	.96
Acetate: propionate ratio	4.04	4.44	0.61	.13

^a^ SS group: TMR with 40% single corn silage, MS group: TMR with various silage (27% corn silage, 10% alfalfa silage, and 15% oat silage).

### 3.4 Diversity of ruminal bacterial communities

**FIGURE 2:**
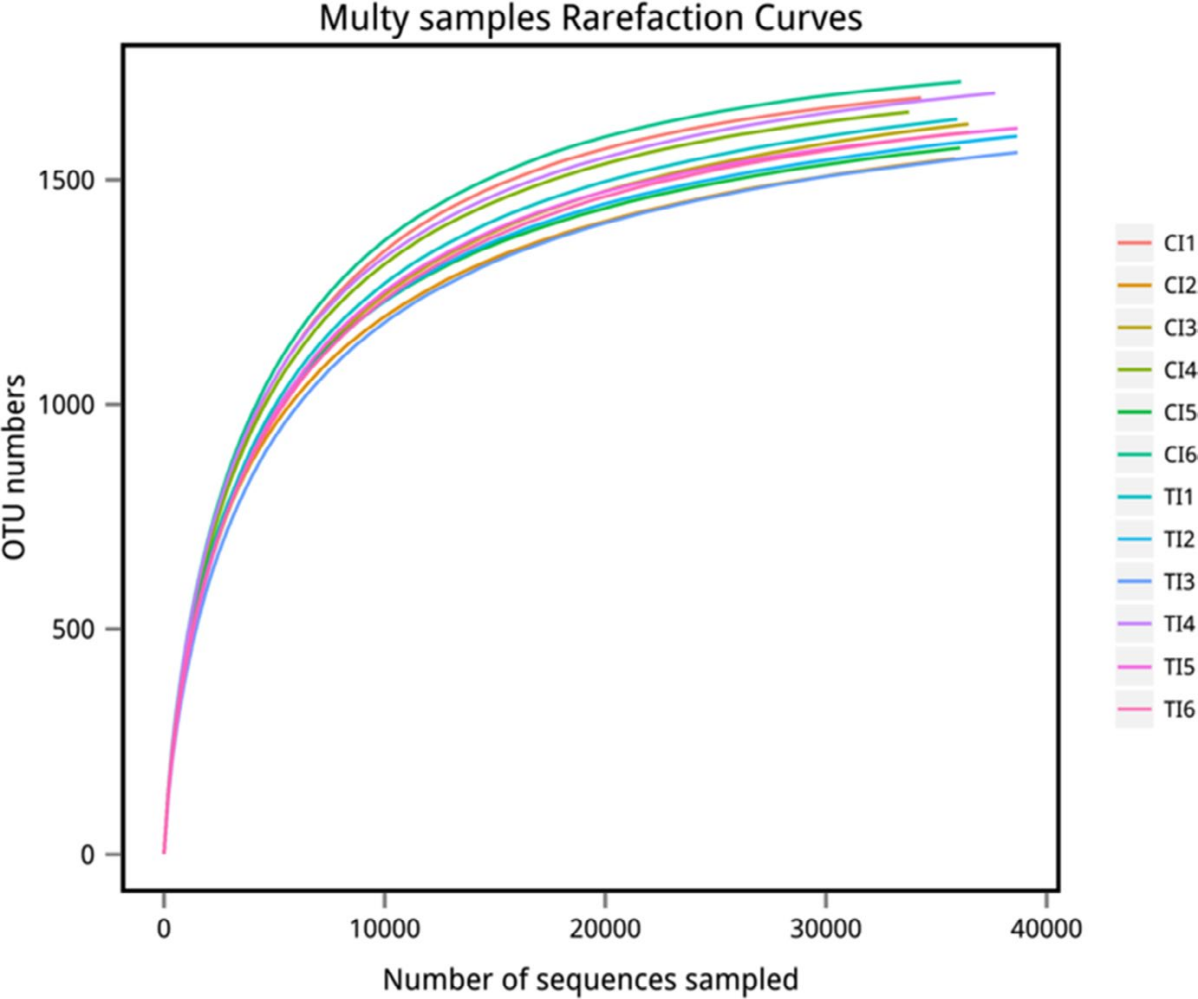
Rarefaction curves obtained from ruminal digesta‐associated microbiome samples of beef cattle fed total mixed ration with various forage silage. CI: Represents the six samples of the SS group; TI: Represents the six samples of the MS group

A total of 958,947 reads were obtained from the 12 samples, and a total of 651,435 Clean tags were generated after the two‐end reads were spliced and filtered, with an average of 54,286 Clean tags obtained from each sample. It was identified that 1,902 OTUs in total in all samples were based on 97% similarity. A total of 18 phyla, 32 classes, 44 orders, 62 families, and 131 genera were identified in the two groups in cattle rumen. Rarefaction curve reflected sufficient sequencing volume (Figure 2).

There were no significant differences in the OTUs, ACE, and Chao1 indices in the MS and SS groups (*p* > .05). But, the Shannon index of the MS group was higher than the SS group (*p* < .05), and the Simpson index of the MS group tended to be higher than the SS group (*p* = .10) (Table 5).

**TABLE 5:** Species richness and alpha‐diversity indexes of the bacterial community (at 97% similarity level)

Item	Groups^a^		*SEM*	*p‐*Value
	SS	MS		
OTU	1,617.83	1,632.33	15.51	.66
ACE	1,707.47	1,718.16	12.07	.68
Chao1	1,736.53	1,733.78	11.63	.91
Simpson	0.008	0.006	0.001	.10
Shannon	6.03	6.21	0.01	.04
Coverage, %	99.50	99.49	0.02	.80

^a^ SS group: TMR with 40% single corn silage, MS group: TMR with various silage (27% corn silage, 10% alfalfa silage and 15% oat silage).

**FIGURE 3:**
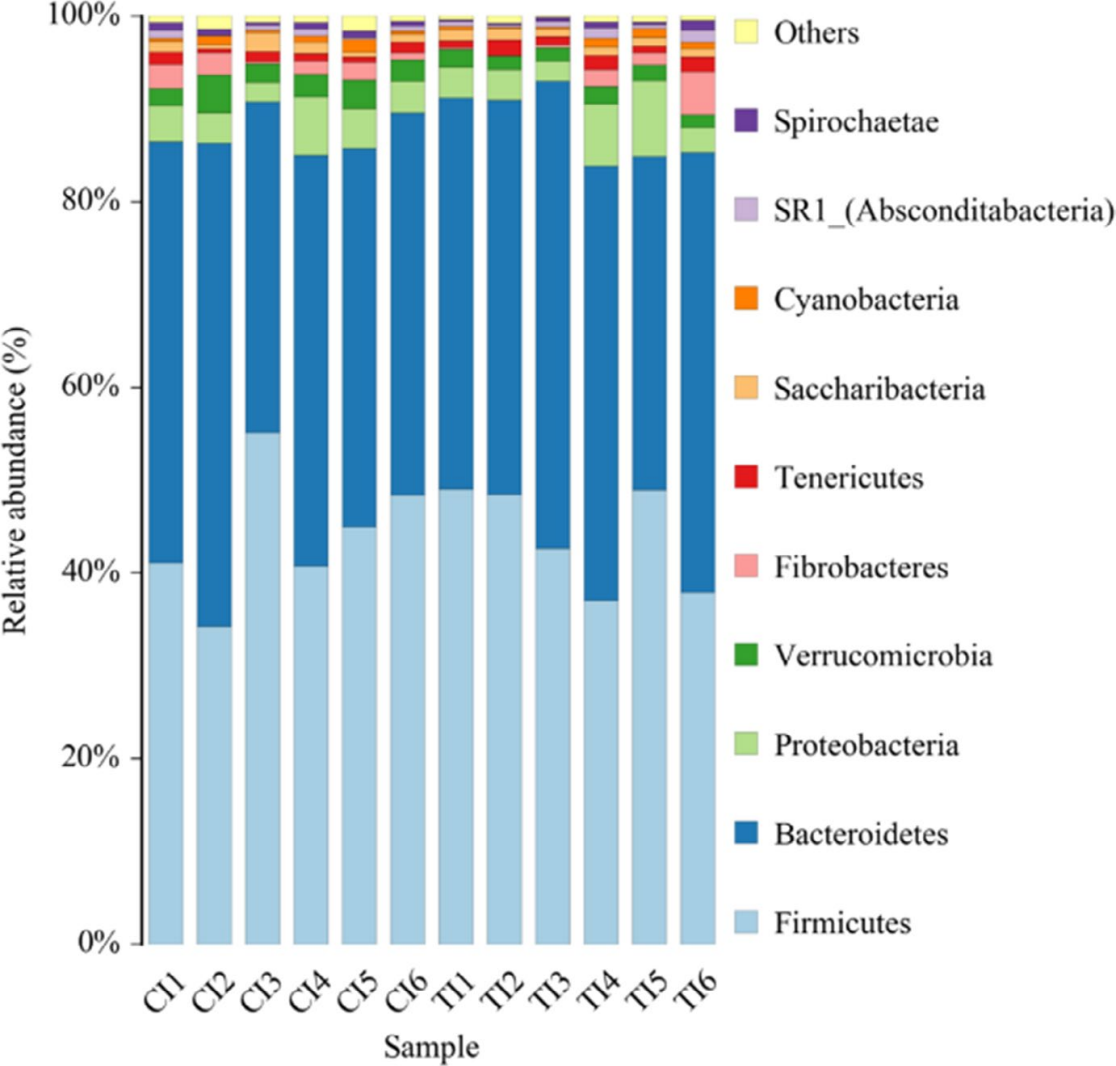
Effects of total mixed ration with various forage silage on bacterial abundance in phylum level. CI: Represents the six samples of the SS group; TI: Represents the six samples of the MS group

**FIGURE 4:**
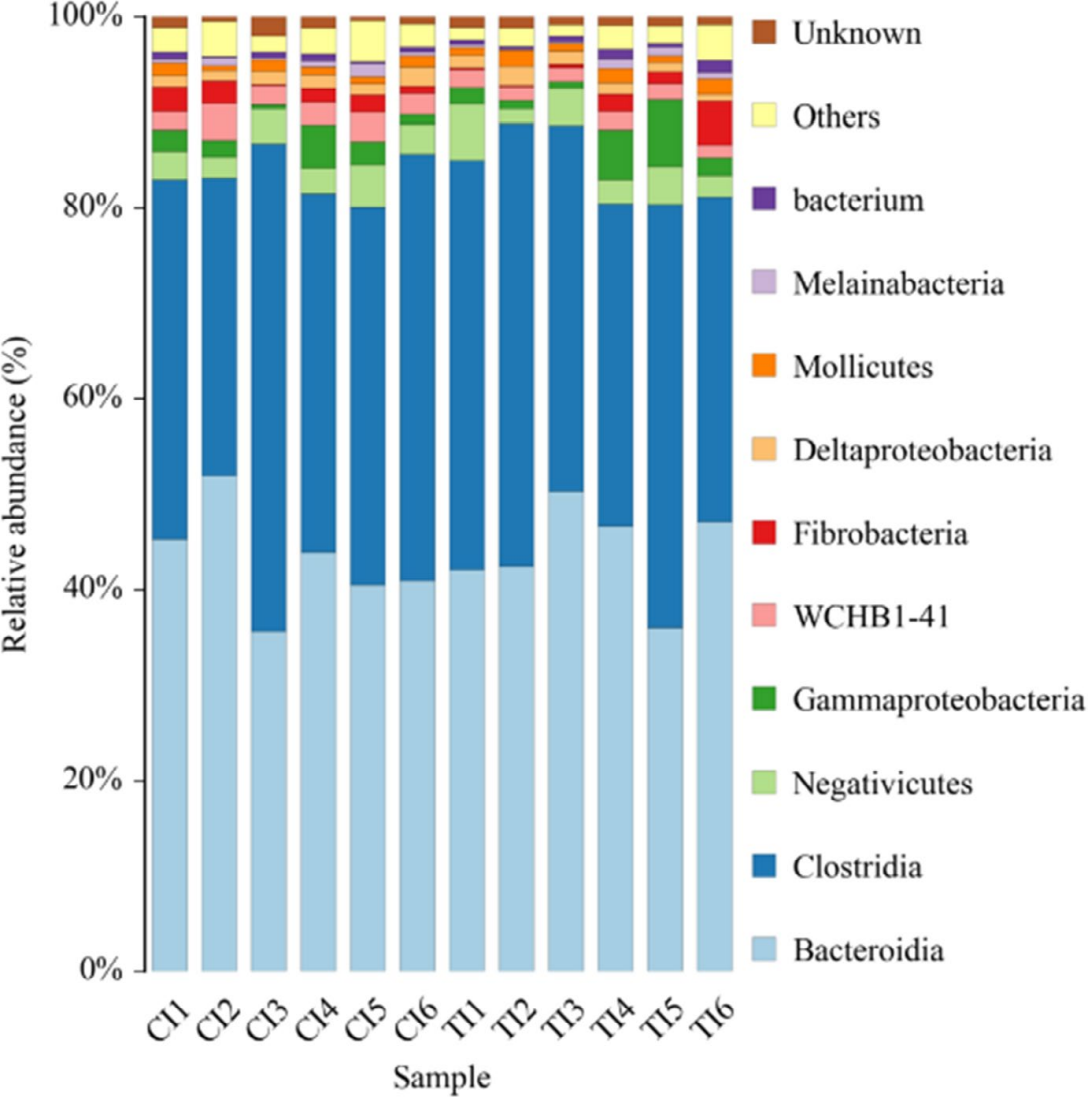
Effects of total mixed ration with various forage silage on bacterial abundance in class level. CI: Represents the six samples of the SS group; TI: Represents the six samples of the MS group

At the phylum level (Table 6 and Figure 3), *Bacteroidetes* and *Firmicutes* were the dominant phyla, occupying 87.80% of all bacteria (44.10% and 43.70%, respectively). A higher *Bacteroidetes/Firmicutes* was found in the MS group than the SS group (*p* < .05). And more, the abundances of the *Verrucomicrobia* (1.63% vs. 2.62%, *p* = .01), *Acidobacteria* (0.03% vs. 0.21%, *p* < .05) and *Chloroflexi* (0.02% vs. 0.07%, *p* = .01) phyla were higher in the MS group than the SS group. However, the abundances of *Firmicutes* (44.00% vs. 44.10%), *Bacteroidetes* (43.20% vs. 44.20%), *Proteobacteria* (3.89% vs. 4.38%), *Fibrobacteres* (1.40% vs. 1.52%), and *Saccharibacteria* (1.00% vs. 1.03%) did not show any significant difference between the two groups. At the class level (Table 6 and Figure 4), *Bacteroidia* had the highest abundance in the SS and MS groups (44.10% and 43.00%), and *Clostridia* was second abundant in the SS and MS groups (40.00% and 40.30%). The abundance of *WCHB1_41* was higher in the MS group (2.54%) than the SS group (1.60%) (*p* = .01).

**TABLE 6:** Effects of TMR with various forage silage on bacterial abundance in phylum and class level (%)

Item	Groups^a^		*SEM*	*p*‐Value
	SS	MS		
*Firmicutes*	44.05	44.10	2.45	.93
*Clostridia*	40.00	40.30	2.20	.92
*Bacteroidetes*	42.60	44.20	2.14	.74
*Bacteroidia*	44.10	43.00	2.06	.74
*Proteobacteria*	3.89	4.38	0.69	.67
*Gammaproteobacteria*	2.91	2.19	1.06	.52
*Alphaproteobacteria*	0.12	0.29	0.03	.09
*Verrucomicrobia*	1.63	2.62	0.13	.01
*WCHB1_41*	1.60	2.54	0.10	.01
*Fibrobacteres*	1.40	1.52	0.54	.85
*Fibrobacteria*	1.40	1.52	0.70	.87
*Saccharibacteria*	1.00	1.03	0.13	.88
*Melainabacteria*	0.55	0.64	0.15	.70
*Acidobacteria*	0.03	0.21	0.02	<.001
*Actinobacteria*	0.09	0.16	0.05	.39
*Chloroflexi*	0.02	0.07	0.01	.01
*Chloroplast*	0.02	0.008	0.01	.07
*Bacteroidetes/Firmicutes*	0.96	1.00	0.02	.04

^a^ SS group: TMR with 40% single corn silage, MS group: TMR with various silage (27% corn silage, 10% alfalfa silage and 15% oat silage).

**FIGURE 5:**
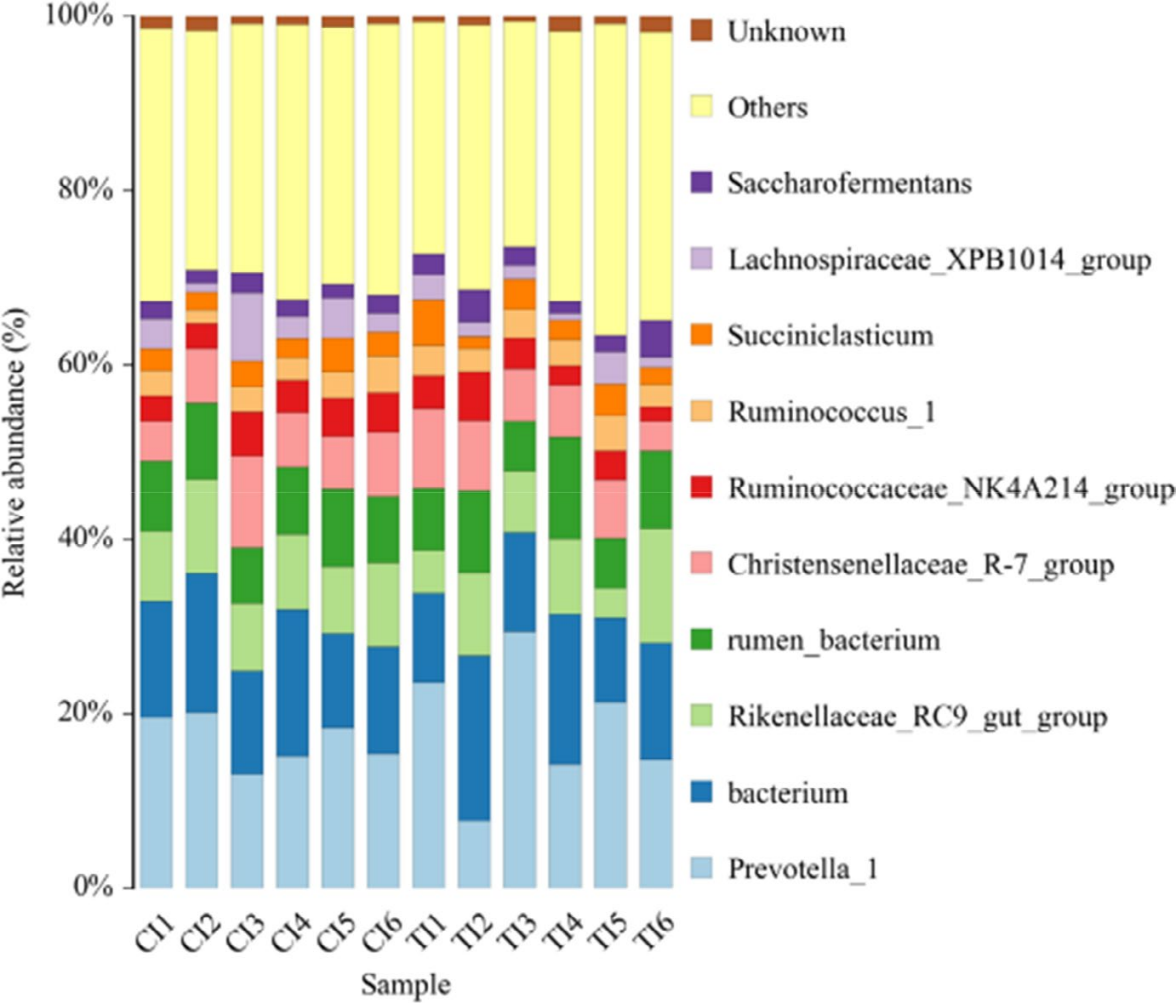
Effects of total mixed ration with various forage silage on bacterial abundance in genus level. CI: Represents the six samples of the SS group; TI: Represents the six samples of the MS group

At the genus level (Table 7 and Figure 5), the 15 genera >1%. The percentage of the genus *Ruminococcus_1* was higher in the MS group than the SS group (*p* = .05). It was found that the dominant genus was *Prevotella_1* accounting for 18.50% in the MS group versus 16.90% in the SS group of total bacteria. Furthermore, the abundances of *Prevotella_1* and *Ruminococcaceae_UCG_014* had an increase in the MS group compared with the SS group (*p* = .04 and *p* = .06).

**TABLE 7:** Effects of TMR with various forage silage on bacterial abundance at genus level (%)

Item	Groups^a^		*SEM*	*p*‐Value
	SS	MS		
*Prevotella_1*	16.90	18.50	0.18	.04
*Rikenellaceae_RC9_gut‐group*	7.78	8.72	1.10	.57
*Christensenellaceae_R−7_group*	6.55	6.84	0.78	.82
*Ruminococcaceae_NK4A214_group*	3.35	3.92	0.46	.44
*Lachnospiraceae_XPB1014_group*	1.97	3.61	0.67	.16
*Ruminococcus_1*	2.84	3.19	0.28	.05
*Succiniclasticum*	2.96	2.71	0.46	.73
*Saccharofermentans*	2.61	1.90	0.26	.17
*Ruminococcaceae_UCG_014*	1.64	2.22	0.20	.06
*Papillibacter*	1.38	1.57	0.16	.52
*Fibrobacter*	1.40	1.52	0.46	.88
*Butyrivibrio_2*	1.48	1.67	0.32	.74
*Prevotellaceae_UCG_001*	1.18	1.34	0.11	.31
*Lachnospiraceae_AC2044_group*	1.22	1.32	0.18	.66
*Ruminococcaceae_UCG_010*	0.99	1.14	0.11	.41

^a^ SS group: TMR with 40% single corn silage, MS group: TMR with various silage (27% corn silage, 10% alfalfa silage, and 15% oat silage).

**FIGURE 6:**
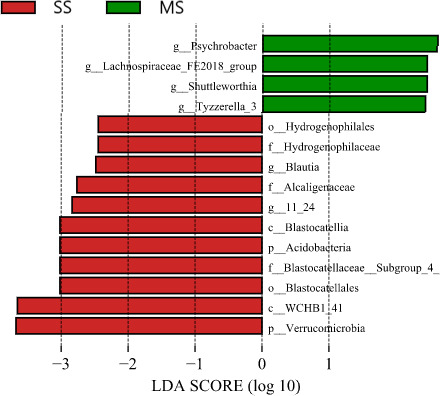
LEfSe analysis showing the taxonomic differences among the two groups in the rumen of beef cattle. All identified taxonomy was significantly different based on LDA score larger than 2.0. MS group: TMR with various silage (27% corn silage, 10% alfalfa silage, and 15% oat silage), SS group: total mixed ration (TMR) with 40% single corn silage

The 15 bacterial taxa identified by LEfSe were significantly enriched in the rumen of different treatment, with 4 (*Sychrobacter*, *Shuttleworthia*, *Tyzzerrella‐3*, etc.) were higher in the MS group, 11 (*Hydrogenophilaceae*, *Blautia*, *Alcaligenaceae*, *Blastocatellia*, *Acidobacteria*, *Blastocatellales*, *WCHBI‐41*, *Verrucomicrobia*, etc.) were lower in the SS group (Figure 6).

**FIGURE 7:**
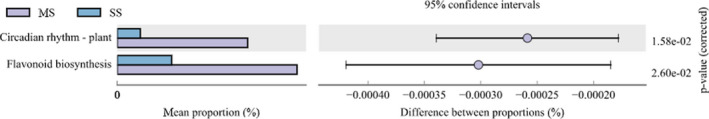
Functional profiles of microbial communities with different diets treatments. The extended error bars show significantly different KEGG pathways between the fractions. MS group: TMR with various silage (27% corn silage, 10% alfalfa silage, and 15% oat silage), SS group: total mixed ration (TMR) with 40% single corn silage

Function prediction is indicated by KEGG analysis in the rumen microbial community with different treatments (Figure 7). The Flavonoid biosynthesis and Circadian rhythm‐plant were higher in the MS group compared with the SS group. Therefore, roughage composition and proportion may be responsible for the significant differences in the microbiome with different treatments. In further studies, it is necessary to compare the changes in the rumen bacteria of long‐term fattened beef cattle.

**FIGURE 8:**
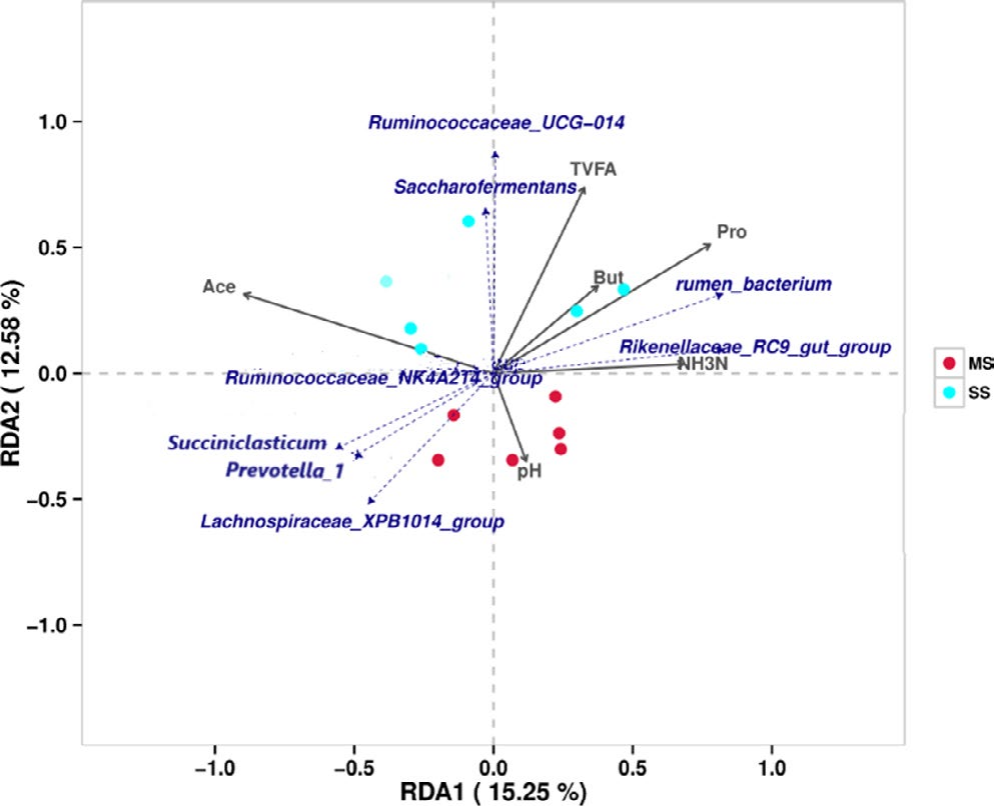
Redundancy analysis of the top 8 known genera in association with six fermentation parameters. Arrows indicate the direction and magnitude of parameters associated with bacterial genera. Ace, Acetate; but, butyrate; pro, propionate; MS group: TMR with various silage (27% corn silage, 10% alfalfa silage, and 15% oat silage), SS group: total mixed ration (TMR) with 40% single corn silage

Redundancy (RDA) analysis showed that two axes explained 27.83% of the differentiation of the microbial community (Figure 8). The arrow length of the environmental factors represents their effect degree on the bacterial genus, and the obtuse angle between environmental factors and bacterial genus indicated that there was a negative correlation between the two groups. The effects of environmental factors on the bacterial genus ranked from high to low were acetate, propionate, TVFA, NH_3_‐N, butyric acid, and pH. There was a positive correlation among the following: acetate concentration with *Ruminococcaceae_NK4A214_group*, *Prevotella_1*; and propionate and TVFA concentrations with *Ruminococcaceae_UCG_014* and *Rikenellaceae_RC9_gut_group*, moreover, the NH_3_‐N concentration with *Rikenellaceae_RC9_gut_group*.

## 4 DISCUSSIONS

As previous studies reported that combination of high‐quality forage can produce a positive combination effect and improve rumen fermentation properties and further elevate the production performance of livestock (Zhang et al., 2015, 2019). In the present study, the diet of the MS group improved the ADFI (5.30%), ADG (16.67%), and reduced FCR (12.76%) of fattening beef cattle compared to the diet of the SS group. The dry matter intake was higher in the MS group compared with the SS group, which could explain the increase of ADG in the MS group compared with the SS group, and also could be explained with the increase in VFA concentration in the MS group, resulting in the increase of metabolizable energy of the diet (Langille et al., 2017).

Blood parameters are an indicator related to pathological diagnosis that reflects the health status of animals exposed to anti‐nutritional or harmful substances (Manoeuvrier et al., 2017). In the present study, serum T‐AOC, SOD, and UN concentrations differed between the two groups, indicating that the antioxidant capacity and protein metabolism of the organism were affected by the roughage ratio and composition. Oxidative stress puts ruminants in sub‐healthy status, thus it is crucial to improve the antioxidant activity of the animal organism. Generally, serum UN is used for diagnosing kidney damage (Cozzi et al., 2011). In the present study, all serum parameters were within normal ranges (Solanas et al., 2010); thus, the difference of UN was mainly due to dietary nutrient composition in the experimental diet, while the proportion of roughage ratio increased in the MS group, the soybean meal and corn amount decreased to balance the dietary crude protein level among diets. However, soybean meal had good protein digestibility (Langille et al., 2017), a decrease might occur in serum UN with a decline in soybean meal proportion.

It was found that the ruminal pH and concentration of NH_3_‐N were not significantly different between the SS and the MS groups, which might be related to rumen environment stability (Duffield et al., 2004; Ellis et al., 2009). Moreover, it also observed that the NH_3_‐N concentration was averagely 14.73 mg/dl (14.88 mg/dl in the SS group vs. 14.57 mg/dl in the MS group). Mcdonald et al. (1995) recommended that the optimum NH_3_‐N concentration range from 8.5 to 30 mg/dl, and if the NH_3_‐N concentration was below the minimum threshold, microbial production and productivity would be inhibited. In this study, the rumen NH_3_‐N concentration of the cattle was within the optimal range.

The VFA is a major carbon source for rumen microbial proliferation (Spears et al., 2004), and provides 60%–80% digestible energy for ruminant (Galyean, 2014). And the composition ratios were important indicators that reflected the rumen digestion and microbial composition (Bergman, 1990). Previous studies showed that higher VFA concentration with equivalent to more efficient energy utilization for animal growth and production (Ozadali et al., 1996). Interestingly, in the present study, the TMR with various silage improved the VFA concentration, implying that the combination of a high‐quality forage diet could improve rumen digestibility and energy conversion by a higher intake of dry matter, and then increase the VFA concentration (Zhou et al., 2012).

The present study data of the higher Shannon index indicated that TMR with multitudinous high‐quality forage would increase the diversity of rumen microbiota. Furthermore, the abundances of *Bacteroidetes* and *Firmicutes* were the highest in the rumen microorganism, which was similar to previous reports in bovine (Bo Trabi et al., 2019), and sheep (Lopes et al., 2015; Perumbakkam et al., 2011), describing the *Bacteroidetes* and *Firmicutes* were dominant phyla in the rumen microbiome. Previous studies had reported that the content of dietary fiber was proportional to the change in *Bacteroidetes/Firmicutes* ratio (Parnell & Reimer, 2012; Trompette et al., 2014). Our present results also showed a higher ratio of *Bacteroidetes/Firmicutes* in the MS group than that the SS group which was relative to higher NDF intake (38.89% vs. 32.77%). Moreover, it was found that a higher *Verrucomicrobia* in the MS group compared with the SS group. *Verrucomicrobia* plays a major role in immune tolerance of the gastrointestinal tract for mammals, and the decrease of species abundance is closely related to the decline of host immunity (Derrien et al., 2011). Therefore, our results indicated that the beef cattle fed TMR with various forage silage could contribute to better health, which corresponds to the results of the previous serum antioxidant parameters. However, the long‐term effects should be further studied.

The genus *Prevotella*, a dominant genus of rumen microorganisms, is involved in the degradation of all dietary components in the rumen (Alzahal et al., 2017; Henderson et al., 2015; Schären et al., 2018). Consistently, the abundance of *Prevotella_1* was greater in the MS group (18.5%) than the SS group (16.9%), indicating that the beef cattle fed TMR with various forage silage was beneficial to the degradation of dietary components in the rumen. *Ruminococcaceae*, is a kind of cellulose‐degrading bacteria, its main products are acetic acid and formic acid (Wood, 1988). In the present study, the higher *Ruminococcaceae_UCG_014* in the MS group was observed by LEfSe analysis compared with the SS group, which could contribute to higher percentage of roughage and fiber content in the MS group. In addition, the specific OTU in *Blautia* was correlated with inflammatory indexes and showed certain anti‐inflammatory effects (Benítez‐Páez et al., 2020), and play an important role in the recovery of intestinal inflammation (Salminen et al., 1998; Trompette et al., 2014). Compared with the SS group, the higher *Blautia* in the MS group was also found by LEfSe analysis, indicating that the MS group diet may help improve intestinal health of beef cattle. However, this is only a hypothesis, their relationship needs further verification.

The KEGG analysis indicated that the flavonoid biosynthesis was higher in the MS group compared with the SS group. The activity of enzymes catalyzed the O_2_‐ production could be inhibited by flavonoids, activated antioxidant enzymes, and thus reduced the production of free radicals (Bao et al., 2016). As a polyphenol, flavonoids endow high antioxidant activity (Kamiloglu et al., 2013). This study detected beef cattle‐fed MS group diets may be beneficial to the synthesis of flavonoids, and may improve the antioxidant function of the animal organism. The RDA analysis revealed that there is a positive relationship between *Prevotella_1* and acetate concentration. Similarly, Henderson et al. (2015) observed that *Prevotella_1* was positively correlated with acetate and pH value. In agreement with our results, Biddle et al. (2013) found *Ruminococcaceae_RC9_gut_group* had a positive correlation with NH_3_‐N concentration.

## 5 CONCLUSIONS

In summary, the beef cattle fed TMR with a variety of silage increased the concentration of TVFAs and individual acids, improved rumen fermentation, average daily weight gain, and feed effectiveness, moreover, improved serum T‐AOC and SOD activities. The species involved in the degradation of dietary components (*Prevotella_1*) and immune tolerance of the gastrointestinal tract (*Verrucomicrobia*) were higher in diet with a variety of silage compared with the single silage. Therefore, the present findings suggest that it is economical and healthy to substitute various silage forage +low level of concentrate for relative single silage forage +high level of concentrate in a finishing diet of beef cattle. Furthermore, the effect of various silage supplementation on production performance and the livestock products quality should be continuously studied for long‐term feeding.

## CONFLICT OF INTEREST

We certify that there is no conflict of interest with any financial organization regarding the material discussed in the manuscript.

## AUTHOR CONTRIBUTIONS

**Xia Zhang:** Conceptualization (equal); Data curation (lead); Formal analysis (lead); Investigation (equal); Methodology (equal); Resources (equal); Software (equal); Supervision (equal); Validation (equal); Visualization (equal); Writing‐original draft (lead); Writing‐review & editing (lead). **Hucheng Wang:** Conceptualization (lead); Data curation (equal); Formal analysis (equal); Funding acquisition (lead); Investigation (equal); Methodology (equal); Project administration (lead); Resources (equal); Software (equal); Supervision (lead); Validation (equal); Visualization (equal); Writing‐review & editing (supporting). **Xusheng Guo:** Conceptualization (equal); Funding acquisition (supporting); Investigation (supporting); Project administration (supporting); Resources (supporting); Software (equal); Supervision (supporting); Validation (equal); Visualization (equal).

## ETHICS STATEMENT

All the animal experimental procedures adhered to the Guidelines for the Care and Use of Laboratory Animals (Ministry of Science and Technology of the People’s Republic of China) and were approved by the Ethical Committee for Animal Experiments, Lan Zhou University.

## Data Availability

All authors confirm that the data supporting the findings of this study are available within the article.
